# Ginger Phytochemicals Inhibit Cell Growth and Modulate Drug Resistance Factors in Docetaxel Resistant Prostate Cancer Cell

**DOI:** 10.3390/molecules22091477

**Published:** 2017-09-05

**Authors:** Chi-Ming Liu, Chiu-Li Kao, Yu-Ting Tseng, Yi-Ching Lo, Chung-Yi Chen

**Affiliations:** 1 Department of Nursing, Tzu Hui Institute of Technology, Pingtung County 92641, Taiwan; beagleliu@gmail.com (C.-M.L.); kkjj77677@gmail.com (C.-L.K.); 2 Department of Pharmacology, School of Medicine, Kaohsiung Medical University, Kaohsiung 80708, Taiwan; mmship1112@yahoo.com.tw; 3 Graduate Institute of Medicine, College of Medicine, Kaohsiung Medical University, Kaohsiung 80708, Taiwan; 4 School of Medical and Health Sciences, Fooyin University, Ta-Liao District, Kaohsiung 83102, Taiwan

**Keywords:** ginger, drug resistance, prostate cancer

## Abstract

Ginger has many bioactive compounds with pharmacological activities. However, few studies are known about these bioactive compounds activity in chemoresistant cells. The aim of the present study was to investigate the anticancer properties of ginger phytochemicals in docetaxel-resistant human prostate cancer cells in vitro. In this study, we isolated 6-gingerol, 10-gingerol, 4-shogaol, 6-shogaol, 10-shogaol, and 6-dehydrogingerdione from ginger. Further, the antiproliferation activity of these compounds was examined in docetaxel-resistant (PC3R) and sensitive (PC3) human prostate cancer cell lines. 6-gingerol, 10-gingerol, 6-shogaol, and 10-shogaol at the concentration of 100 μM significantly inhibited the proliferation in PC3R but 6-gingerol, 6-shogaol, and 10-shogaol displayed similar activity in PC3. The protein expression of multidrug resistance associated protein 1 (MRP1) and glutathione-S-transferase (GSTπ) is higher in PC3R than in PC3. In summary, we isolated the bioactive compounds from ginger. Our results showed that 6-gingerol, 10-gingerol, 6-shogaol, and 10-shogaol inhibit the proliferation of PC3R cells through the downregulation of MRP1 and GSTπ protein expression.

## 1. Introduction

Prostate cancer is the most prevalent cancer in the western countries. Prostate cancer can be diagnosed in the early stage by detection of specific antigen called prostate-specific antigen (PSA) [1,2,3]. Prostate cancer can be divided into two types of prostate cancers including hormone-dependent and castration-resistant prostate cancer [4]. Initially, prostate cancer is sensitive to hormonal therapy. However, resistance to androgen deprivation therapy ultimately occurs in the patients [5]. It should be noted that androgen-independent prostate cancer is often insensitive to anti-cancer drugs [6,7,8]. Prostate cancer cell is resistant to chemotherapy because of escape from apoptosis [9]. Drug resistance is a critical issue for the cancer therapy, but the underlying mechanisms are not clear.

A lot of studies have been proposed to contribute to drug resistance in tumor cells. Multidrug resistance (MDR) mechanisms are associated with increased expression of the P-gp (P-glycoprotein) or increased cellular metabolism of drug detoxifying proteins, such as glutathione-S-transferase (GST) [10,11]. In particular, elevated P-gp or GST are associated with increased resistance to apoptosis. Recently, more and more studies have focused on natural products with P-gp or GST inhibition in cancer cells [12]. Multidrug resistance associated protein 1 (MRP1) also plays an important role in the development of drug resistance in many cancers. MRP1 is overexpressed in many chemoresistance cancer types including gastric cancer, neuroblastoma, and prostate cancer [13,14,15,16].

Ginger belongs to Zingiberaceae family and popular used in Asia countries. Ginger has been used as a spice and herbal medicine in China over a thousand years. Ginger is widely used in many diseases including diarrhea, nausea, gastritis, and cancer [17,18]. Studies have shown that 6-gingerol, 8-gingerol, 6-dehydrogingerdione, 6-shogaol, and 10-shogaol, are bioactive compounds form ginger [19,20]. In our previous studies, we have demonstrated that 6-dehydrogingerdione induced cell proliferation and migration [21]. Moreover, we also found that 6-gingerol can disturb the Ca^2+^ in Madin-Darby canine kidney cells [22].

**Figure 1:**
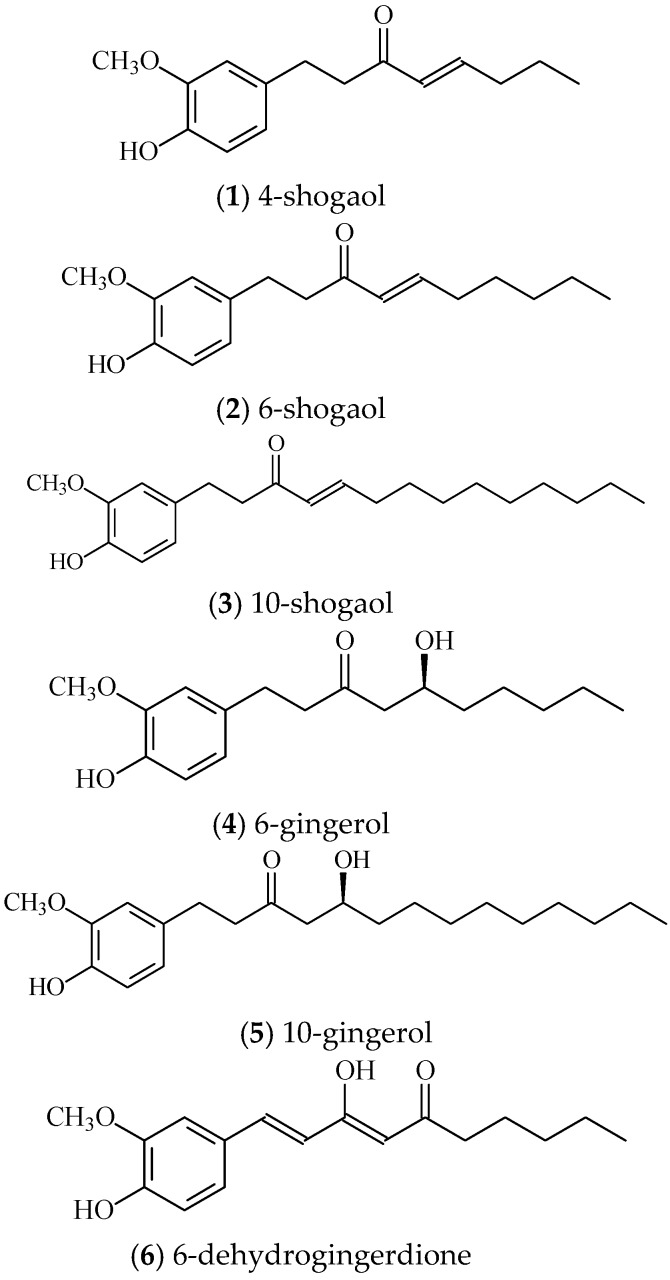
The chemical structure of 4-shogaol (**1**), 6-shogaol (**2**), 10-shogaol (**3**), 6-gingerol (**4**), 10-gingerol (**5**), and 6-dehydrogingerdione (**6**).

Studies have shown that 6-gingerol, 8-gingerol, 10-gingerol, and 6-shogaol, have anti-cancer activity in prostate cancer cell line [23,24]. Plant-based functional foods rich in different phytochemicals with supposed additive or synergic effects in the various mechanisms of actions have recently showed considerable anti-tumor effects using in vitro and in vivo cancer models [25]. However, few studies have examined the anti-cancer activity of ginger phytochemicals in drug resistant prostate cancer cells. In the present study, we investigate the relative cytotoxic potencies of ginger phytochemicals including 4-shogaol, (**1**), 6-shogaol, (**2**), 10-shogaol (**3**), 6-gingerol (**4**), 10-gingerol (**5**), and 6-dehydrogingerdione (**6**) (Figure 1) in a docetaxel resistant prostate cancer cell line. In addition, we also examined whether these compounds disturb multidrug resistance protein expression in docetaxel resistant prostate cancer cell line.

## 2. Results

### 2.1. Docetaxel Resistant PC3 Cell

#### Docetaxel Increases GSTπ and MRP1 Protein Expression in Prostate Cancer Cell Line

**Figure 2:**
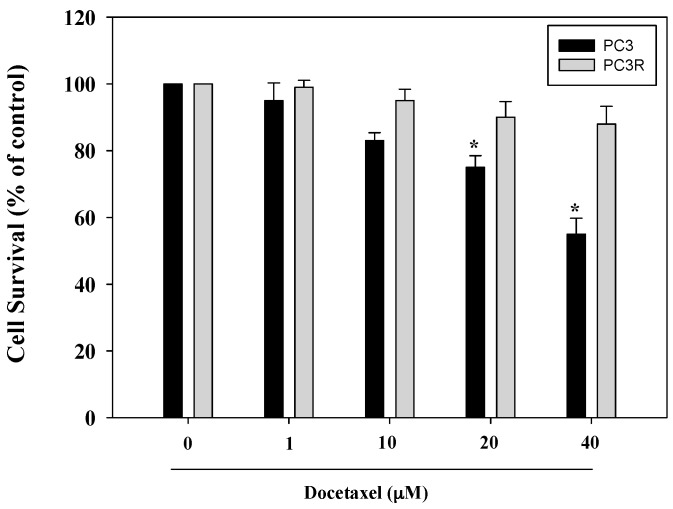
Effects of docetaxel on PC3 and PC3R cell viability at 24 h time point. Data were summarized from three independent experiments. * *p* < 0.05 vs. docetaxel (0 μM) group.

**Figure 3:**
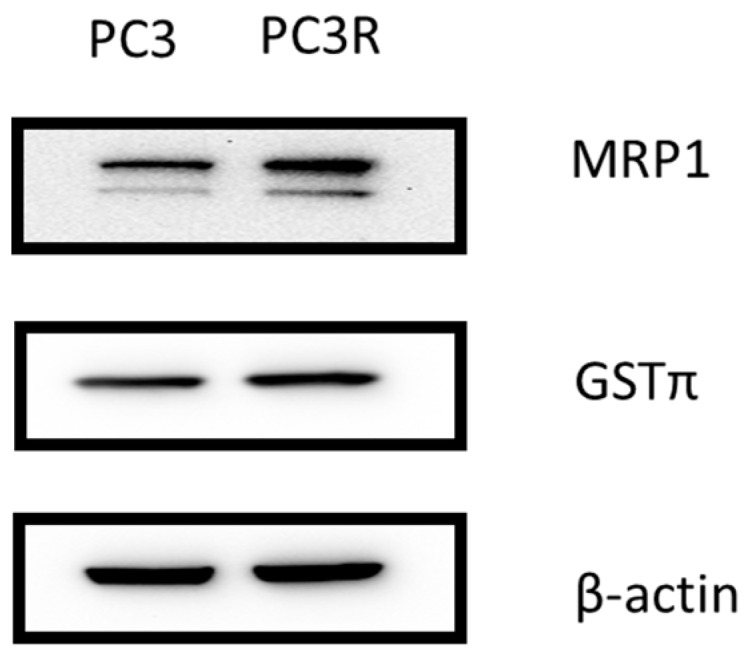
The protein expression of multidrug resistance associated protein 1 (MRP1) and glutathione-S-transferase (GSTπ) in docetaxel-resistant (PC3R) and PC3 human prostate cancer cells. Results are representative of three experiments.

PC3 is a castration-resistant prostate cancer cell line. The docetaxel-resistant cell line, PC3R, was developed through serial passage of PC3 with docetaxel treatments as previously described with modification [26]. In the current study, the cell viability of PC3 with docetaxel treatments were 95 ± 5.3%, 83 ± 2.4%, 75 ± 3.5%, and 55 ± 4.8% (1, 10, 20, 40 μM) for 24 h. The IC_50_ value for docetaxel was 42 ± 3.4 μM in the PC3 cells. The sequence of docetaxel (1, 10, 20, 40 μM) treatments showed maximum 10% inhibition in PC3R cells (Figure 2). Further, we compared the GSTπ and MRP1 protein expression in PC3 and PC3R. In our study, the expression of MDR1 is barely detectable in PC3 and PC3R cell lines (data not shown). The expression of MRP1 and GST is slightly increased by docetaxel. The results were shown in Figure 3.

### 2.2. The Pharmacological Activities of Ginger Phytochemicals

#### 2.2.1. Ginger Phytochemicals Inhibit Prostate Cancer Cell Proliferation

**Figure 4:**
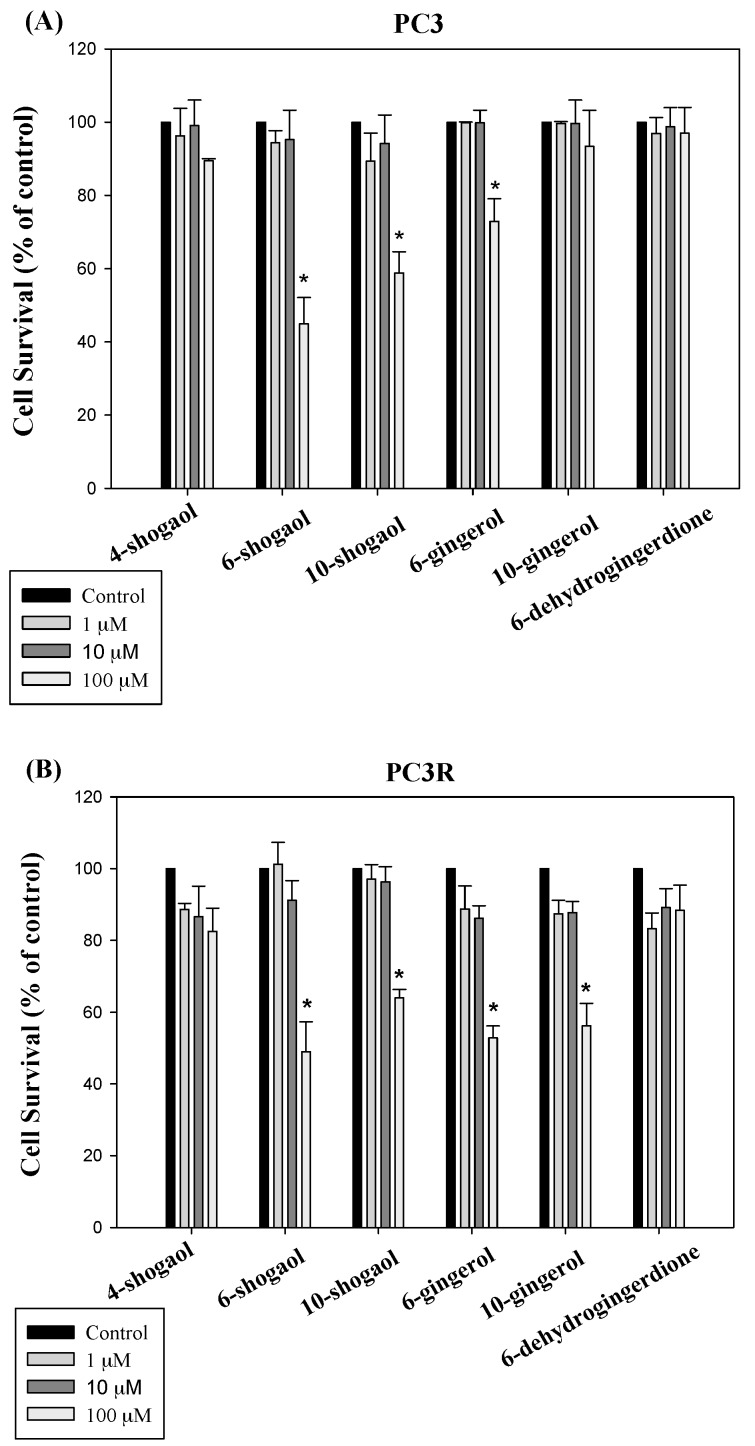
Effects of 4-shogaol (**1**), 6-shogaol (**2**), 10-shogaol (**3**), 6-gingerol (**4**), 10-gingerol (**5**), and 6-dehydrogingerdione (**6**) on PC3 (**A**) and PC3R (**B**) cell viability. These compounds decreased cell viability at 24 h time point. Data were summarized from three independent experiments. * *p* < 0.05 vs. control group.

The major bioactive compounds of ginger are gingerol, shogaol, and dehydrogingerdione. Thus, we determined the anti-proliferation of these compounds including 4-shogaol (**1**), 6-shogaol (**2**), 10-shogaol (**3**), 6-gingerol (**4**), 10-gingerol (**5**), and 6-dehydrogingerdione (**6**) in PC3 and PC3R cell lines. 6-shogaol (**2**), 10-shogaol (**3**), 6-gingerol (**4**), and 10-gingerol (**5**) significantly inhibited proliferation at the concentration of 100 μM for 24 h in PC3R. However, 6-shogaol (**2**), 10-shogaol (**3**), and 6-gingerol (**4**) significantly inhibited proliferation at the concentration of 100 μM in PC3. These results were shown in Figure 4.

#### 2.2.2. Ginger Phytochemicals Disturbed GST and MRP-1 Protein Expression in PC3R Cells

**Figure 5:**
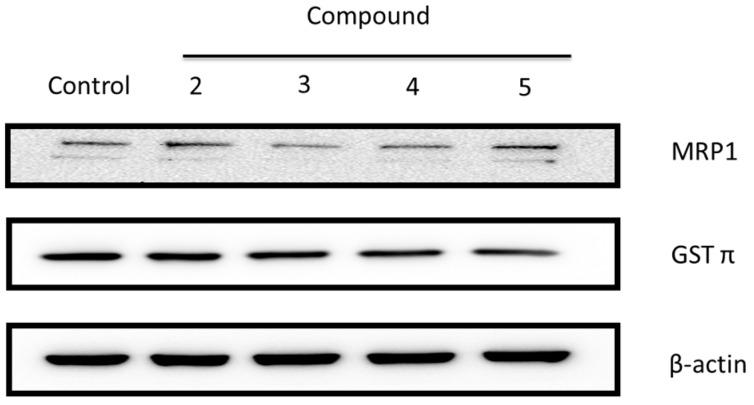
Effects of 6-shogaol (**2**), 10-shogaol (**3**), 6-gingerol (**4**), and 10-gingerol (**5**) (100 μM) on MRP1 and GSTπ in PC3R at 24 h time point. Results are representative of three experiments.

We found that GSTπ and MRP1 protein expression in PC3R cell was higher than in PC3 cells. We further examined the protein level of GSTπ and MRP1 at 100 μM of 6-shogaol (**2**), 10-shogaol (**3**), 6-gingerol (**4**), and 10-gingerol (**5**) treatment at 24 h in PC3R cells. As shown in Figure 5, the protein expression was decreased after treatment. These results shown that 6-shogaol (**2**), 10-shogaol (**3**), 6-gingerol (**4**), and 10-gingerol (**5**) can affect GSTπ and MRP-1 protein expression in docetaxel resistant PC3 cell line (PC3R). The results were shown in Figure 5.

## 3. Discussion

Prostate cancer is a common malignancy and cause of death in western countries. An early diagnosis contributes to the cure of prostate cancer, but many patients still have metastatic disease. Chemotherapy is typical treatment for prostate cancer patient. For prostate cancer, the first chemo drug is docetaxel combined with the steroid drug [27]. Chemotherapy has severe side effects on humans and largely ineffective in progression prostate cancer. Surgical castration, oral estrogen, antiandrogen therapy, etc., are treatments for metastatic prostate cancer. However, prostate cancer often become refractory to hormone therapy. At this stage, the prostate cancer is resistant to chemical drugs. It is important to investigative the mechanism of action of anti-cancer drug resistance for the treatment of prostate cancer. A lot of studies have shown that bioactive food components such as polyphenol, curcumin, flavonoids, and lycopene have chemopreventive activity [24,28,29,30,31]. The results of this study have shown that 6-gingerol, 10-gingerol, 6-shogaol, and 10-shogaol exert significantly inhibitory effect in PC3 and PC3R cell lines at the concentration of 100 μM. PC3 cells are not sensitive to androgen and have high metastatic potential compared to LNCaP and DU145 cells. Thus, the antiproliferation activity of 6-gingerol, 10-gingerol, 6-shogaol, and 10-shogaol are effective at high concentration in both cell lines.

The mechanism of action of drug resistance in cancer cell is complex. Drug resistance is largely mediated through many factors including overexpression of drug resistance proteins. P-glycoproteins are encoded by the MDR gene family and associated with major obstacle in cancer chemotherapy. In humans, MDR1 is involved in transport of many antitumor agents. GST is a well-known family of Phase II detoxification enzyme. The alpha, Mu and Pi class the most studied in cellular resistance [32]. Studies shown that elevated levels of GSTπ are associated with anti-apoptosis and drug resistance. Multidrug resistance-associated protein 1 (MRP1/ABCC1) is a member of the ATP-binding cassette (ABC) transporter superfamily. MRP1 is also expressed in normal tissue but many studies have shown upregulation of MRP1 in many solid tumors [16]. MRP1 is also associated with exporting of endogenous intermediates from toxic insult. MRP1 mediated drug efflux to increase of GSH.

A study has shown that MRP and GSTπ is expressed in PC3 cell line, but P-gp is not expressed [33]. One study reported that the expression of P-gp is expressed in PC3 and DU145 cell lines [34]. Multidrug resistance cell lines are derived by stepwise selection with a single chemotherapy drug. Moreover, MRP1 overexpression confers chemoresistance in prostate cancer line treated with doxorubicin [31]. In this study, multidrug drug resistance prostate cancer cell line PC3R is established from parent prostate cancer cell line PC3 by selection in docetaxel treatment. We conclude that MRP1 and GSTπ is upregulated in PC3R cell line but MDR1 is barely detectable in PC3 and PC3R cell lines.

Studies have shown that ginger and its bioactive compounds can induce apoptosis and inhibit proliferation in many cancer cells including prostate, liver, colon, and so on. The active ingredient of ginger, 6-gingerol, can target cellular molecules and inhibit proliferation, invasion and angiogenesis. 6-gingerol disturb Rb, MAPK, PI3K/Akt, ERK, cyclin A, and CDK expression [35,36]. Shogaol can modulate COX-2, MAPK, PI3K/Akt, cyclin D1, MMP-9, and caspase expression [37]. Few studies investigate ginger and its bioactive compounds in multidrug drug resistance cancer cell.

Our present study provided novel data and demonstrated that ginger phytochemicals display anti-proliferation in chemoresistant prostate cancer cell line. A lot of studies have shown that ginger and ginger extractions displayed chemoprevention activities on in vivo and in vitro studies. However, few studies discussed about the detail pharmacological activities in clinical studies. At least, the current study indicated that these ginger phytochemicals can modulate the MRP and GSTπ protein expression. In the future, we will further examine these phytochemicals for sensitizing the chemotherapy drug activity in chemoresistance cancer cells.

## 4. Materials and Methods

### 4.1. Extraction and Isolation

#### Plant Material

The rhizomes of *Z. officinale* were chipped and air dried and extracted repeatedly with MeOH (10 L × 5) at room temperature. The combined MeOH extracts (530.1 g) were then evaporated and further separated into three fractions by column chromatography on silica gel (70–230 mesh) with gradients of CH_2_Cl_2_/MeOH. Part of fraction 1 (18.6 g) was subjected to silica gel chromatography by eluting with *n*-hexane-acetone (50:1), enriched with acetone to furnish four further fractions (1-1–1-4). Fraction 1-1 (5.2 g) was further purified on a silica gel column using *n*-hexane/acetone mixtures to obtain [4]-shogoal (36 mg) and [6]-dehydrogingerdione (18 mg). Fraction 1-2 (7.4 g) was subjected to silica gel chromatography by eluting with *n*-hexane-acetone (40:1), enriched with acetone to furnish four further fractions (1-2-1–1-2-4). Fraction 1-2-1 (2.8 g) was further purified on a silica gel column using *n*-hexane/acetone mixtures to obtain [6]-gingerol (42 mg) and [10]-shogoal (46 mg). Fraction 1-2-2 (3.2 g) was further purified on a silica gel column using *n*-hexane/acetone mixtures to obtain [6]-shogoal (26 mg) and [10]-gingerol (55 mg).

### 4.2. Pharmacological Assay

#### 4.2.1. Cell Culture

The human prostate carcinoma cell line PC-3 were obtained from Food Industry Research and Development Institute (FIRDI; Hsinchu, Taiwan). PC-3 cells were cultured in F-12 (Gibco) with 10% FBS under standard culture conditions.

#### 4.2.2. Establishment of Drug Resistance Prostate Cell Line

Drug resistance prostate cell line was modified by previous method [26]. Briefly, PC3R was selected for resistance to docetaxel by a stepwise increase in the concentration of docetaxel from 0.1 nM to 40 nM in PC3 cell. The cells were stable in each new concentration and finally reach to 40 nM of docetaxel. As the cells adapted to the concentration, the drug concentration was increased. It took an average three weeks for the cells to be stable in the new concentration. The PC3R cell line was named according to the highest concentration.

#### 4.2.3. Cell Proliferation Assay

After cell attachment, the medium was removed and replaced by either a medium containing different concentration of 6-gingerol, 10-gingerol, 4-shogaol, 6-shogaol, 10-shogaol, and 6-dehydrogingerdione. The 3-[4,5-dimethylthiazol-2-yl]-2,5-diphenyltetrazolium bromide (MTT; Sigma–Aldrich, Co., St. Louis, MO, USA) assay was performed to count cell proliferation.

#### 4.2.4. Western Blot

After incubation, cells were extracted by using protein extraction reagent (Pierce Biotechnology, Rockford, IL, USA). Twenty micrograms of protein was used in SDS—polyacrylamide gel electrophoresis and electrophoretically transferred onto nitrocellulose blots. The nitrocellulose blots were incubated with an anti-MDR1, anti-GSTπ, and anti-MRP1 (Cell Signaling Technology, Inc., Danvers, MA, USA) antibody. The membranes were incubated with horseradish peroxidase-conjugated antibody against mouse, goat, or rabbit IgG (Santa Cruz Biotechnology, Santa Cruz, CA, USA) for 1 h. Each membrane was developed with the enhanced chemiluminescence for the detection of the specific antigen.

### 4.3. Statistical Analysis

All data are expressed as the mean ± S.E.M. Whenever a control group was compared with more than one treatment group, one-way analysis of variance was used. When the analysis of variance found a statistical difference, results were further analyzed using Dunnett’s test. A *p* value of less than 0.05 was considered significant in all experiments.

## 5. Conclusions

Drug resistance has been serious problem in the treatment of cancer. In the current study, the protein expression of MRP1and GSTπ is increased in docetaxel-resistant human prostate cancer cells. Further, the ginger phytochemicals including 6-gingerol, 10-gingerol, 6-shogaol, and 10-shogaol (100 μM) can significantly inhibit docetaxel-resistant human prostate cancer cells growth and reverse drug resistance protein expression including MRP1 and GSTπ expression. In the future, we will further examine the additive or synergic effects of ginger phytochemicals with chemotherapeutic agent in docetaxel-resistant human prostate cancer.
